# Conditional survival estimate of acute-on-chronic hepatitis B liver failure: A dynamic prediction based on a multicenter cohort

**DOI:** 10.18632/oncotarget.4666

**Published:** 2015-07-15

**Authors:** Ming-Hua Zheng, Sheng-Jie Wu, Ke-Qing Shi, Hua-Dong Yan, Hai Li, Gui-Qi Zhu, Yao-Yao Xie, Fa-Ling Wu, Yong-Ping Chen

**Affiliations:** ^1^ Department of Infection and Liver Diseases, Liver Research Center, The First Affiliated Hospital of Wenzhou Medical University, Wenzhou 325000, China; ^2^ Institute of Hepatology, Wenzhou Medical University, Wenzhou 325000, China; ^3^ Department of Cardiovascular Medicine, The Heart Center, The First Affiliated Hospital of Wenzhou Medical University, Wenzhou 325000, China; ^4^ Department of Infectious Diseases, Ningbo 315010, China; ^5^ Department of Intensive Care Unit, Tianjin Infectious Disease Hospital, Tianjin 300000, China; ^6^ School of the First Clinical Medical Sciences, Wenzhou Medical University, Wenzhou 325000, China; ^7^ Department of Clinical Laboratory, The First Affiliated Hospital of Wenzhou Medical University, Wenzhou 325000, China

**Keywords:** acute-on-chronic hepatitis B liver failure, conditional survival, relative survival, prognosis, risk factor

## Abstract

**Objectives:**

Counseling patients with acute-on-chronic hepatitis B liver failure (ACHBLF) on their individual risk of short-term mortality is challenging. This study aimed to develop a conditional survival estimate (CSE) for predicting individualized mortality risk in ACHBLF patients.

**Methods:**

We performed a large prospective cohort study of 278 ACHBLF patients from December 2010 to December 2013 at three participating medical centers. The Kaplan-Meier method was used to calculate the cumulative overall survival (OS). Cox proportional hazard regression models were used to analyze the risk factors associated with OS. 4-week CSE at “X” week after diagnostic established were calculated as CS_4_ = OS_(X+4)_/OS_(X)_.

**Results:**

The actual OS at 2, 4, 6, 8, 12 weeks were 80.5%, 71.8%, 69.3%, 66.0% and 63.7%, respectively. Using CSE, the probability of surviving an additional 4 weeks, given that the patient had survived for 1, 3, 5, 7, 9 weeks was 74%, 86%, 92%, 93%, 97%, respectively. Patients with worse prognostic feathers, including MELD > 25, Child grade C, age > 45, HE, INR > 2.5, demonstrated the greatest increase in CSE over time, when compared with the “favorable” one (Δ36% vs. Δ10%; Δ28% vs. Δ16%; Δ29% vs. Δ15%; Δ60% vs. Δ12%; Δ33% vs. Δ12%; all *P* < 0.001; respectively).

**Conclusions:**

This easy-to-use CSE can accurately predict the changing probability of survival over time. It may facilitate risk communication between patients and physicians.

## INTRODUCTION

Acute-on-chronic liver failure (ACLF) is a critical clinical entity that occurs in patients with acute deterioration of diagnosed or undiagnosed chronic liver disease. It is accompanied with a high mortality rate ranging from 32% to 68%, which occurs mainly in the first 3 months once diagnosed [1–2]. In areas of high hepatitis B virus (HBV) prevalence, such as the developing countries in Asia, acute-on-chronic hepatitis B liver failure (ACHBLF) accounts for >70% of ACLF and almost 120000 patients die per year [3–5]. Due to the unavailability of accurate, reliable and accessible screening tools for predicting which patients are at a borderline risk of worsening disease with an increased chance of fatal outcome, makes appropriate risk stratification and physician-patient communication challenging. Identifying patients at risk of disease progression to to death may help in early management decisions and in justifying health resource allocation [6].

Recently, considerable effort has been extended to earlier and/or more accurate methods to predict the prognosis of this specific clinical condition [7–11]. Most of the models and/or scoring systems are based on the variable of a single-point measurement, mainly at the point of diagnosis or during patient hospitalization. However, these static variables, invariably taken at a single-point in time may be characterized by poor sensitivity and specificity, especially during the early stages of the disease. A dynamic prediction of disease progression and outcome is urgently required [4, 12].

Survival estimates for ACHBLF are commonly reported as survival time following diagnosis. However, such survival estimates, based on conventional survival curves may not provide a real-time prediction for survival. This is largely due to the fact that the risk of death often is highest during the initial few weeks of follow-up after the date of diagnosis is established. Due to this reason, conditional survival estimate (CSE), which accounts for existing survival time has been proposed as a more relevant way to precisely predict the prognosis [12]. In recent years, CSE has been widely introduced in clinical oncology for clinical validation, including predicting the overall survival (OS) and disease-free survival of gastric cancer after surgical resection [13–15], metastatic renal-cell carcinoma [16–17], lung cancer [18–19], diffuse large B-cell lymphoma [20], pancreatic ductal adenocarcinoma [21] and breast cancer [22]. This type of exploration is in its early stage as the only study reported to date is in the field of cardiac failure [23]. As shown with above studies, CSEs are easy-to-use ladder diagrams for predicting the risk of an individual developing an outcome over a specified time period. Furthermore, CSE may provide a more “dynamic” or “real-time” estimate of the risk of death over time and is significantly different from traditional survival estimates [24]. In turn, CSE can be more helpful in tailoring patient-specific treatment, surveillance, and education based on individual survival characteristics.

The specific aim of the current study was to develop a simple and clinically useful CSE for assessing short-term mortality risk in a multicenter cohort of ACHBLF patients. Moreover, we also assessed the effect of various independent risk factors on OS and CSE among ACHBLF patients.

## RESULTS

### Baseline characteristics

A total of 278 cases were enrolled in this study. During the follow-up of 36.2 ± 12.3 months, 109 patients (39.2%) died. The mean age was 45.8 ± 13.2 years, and the patients were predominantly men (77.3%). The most common complication of ACHBLF was ascites (156 patients; 56.1%) followed by LC (127 patients; 45.6%), SBP (101 patients; 36.6%), HE (57 patients; 20.5%) and HRS (15 patients; 5.4%) (Supplemental Table 1). Patients who survived had a lower MELD score (23.3 vs. 28.9, *P* < 0.001), Child score (8.73 vs. 9.71, *P* < 0.001), INR (2.49 vs. 2.9, *P* = 0.025), creatinine (67.25 μmol/L vs. 84.95 μmol/L, *P* < 0.001), TB (270.38 μmol/L vs. 330.89 μmol/L, *P* < 0.001), alkaline phosphatase (144.6 U/L vs. 166.68 U/L, *P* = 0.002) value and higher albumin (31.43 g/L vs. 29.94 g/L, *P* = 0.032), haemoglobin (127.31 g/L vs. 121.62 g/L, *P* = 0.042) and serum sodium (137.18 mmol/L vs. 134.72 mmol/L, *P* < 0.001) value (Table 1). Moreover, the death group had a significantly higher incidence of HE, LC, SBP, ascites when compared with the survival group (*P* < 0.001 for all, Table 1).

**Table 1:** Characteristics of patients with acute-on-chronic hepatitis B liver failure included in this study, stratified by different outcomes

Variable	Survival group (*n* = 169)	Death group (*n* = 109)	*P*-value
**Clinical parameters**			
Age (years)	42.04 ± 12.29	51.63 ± 12.4	<0.001
Male gender No.(%)*	133 (78.7%)	82 (75.2%)	0.500
Hepatic encephalopathy No.(%)*	12 (7.1%)	45 (41.3%)	<0.001
Liver cirrhosis No.(%)*	54 (32.0%)	73 (67.0%)	<0.001
Infection No.(%)*	45 (26.6%)	56 (51.4%)	<0.001
Ascites No.(%)*	80 (47.3%)	76 (79.7%)	<0.001
Hepatorenal syndrome No.(%)*	7 (4.1%)	8 (7.3%)	0.249
**Laboratory parameters**			
White blood cell (10^12^/L)	7.35 ± 5.01	7.41 ± 4.05	0.910
Haemoglobin (g/L)	127.31 ± 21.96	121.62 ± 23.07	0.042
Platelet (10^9^/L)	45.27 ± 31.07	47.39 ± 28.99	0.569
Serum sodium (mmol/L)	137.18 ± 4.89	134.72 ± 5.67	<0.001
ALT (U/L)	624.21 ± 659.74	540.76 ± 628.16	0.295
AST (U/L)	483.76 ± 536.35	566.26 ± 834.14	0.316
Albumin (g/L)	31.43 ± 5.89	29.94 ± 5.43	0.032
Alkaline phosphatase (U/L)	144.6 ± 47.03	166.68 ± 71.62	0.002
Total bilirubin (μmol/L)	270.38 ± 153.14	330.89 ± 170.34	0.002
Creatinine (μmol/L)	67.25 ± 26.46	84.95 ± 56.55	<0.001
INR	2.49 ± 1.64	2.9 ± 1.22	0.025
**Scoring systems**			
MELD score	23.3 ± 6.39	28.9 ± 8.98	<0.001
Child score	8.73 ± 1.58	9.71 ± 1.4	<0.001

**NOTE:** ALT, alanine aminotranferase; AST, aspartate aminotranferase; INR, international normalized ratio; MELD, model for end-stage liver disease.

* Dichotomous values.

### Factors associated with overall survival

At a mean follow-up of 36.2 ± 12.3 months, OS was 60.8%. Several factors were associated with a worse OS on univariate analysis. Specifically, increasing age (hazard ratio (HR) = 1.045, 95% CI (confidence interval) 1.030–1.060; *P* < 0.001) and HE (HR = 4.610, 95% CI 3.125–6.799; *P* < 0.001) were associated with worse OS. Other risk factors for worse prognosis included LC (HR = 2.930, 95% CI 1.963–4.373; *P* < 0.001), SBP (HR = 2.292, 95% CI 1.573–3.340; *P* < 0.001), ascites (HR = 2.043, 95% CI 1.357–3.077; *P* < 0.001), serum sodium (HR = 1.000, 95% CI 1.000–1.001; *P* = 0.030), AST (HR = 4. 610, 95% CI 3.125–6.799; *P* < 0.001), alkaline phosphatase (HR = 1.002, 95% CI 1.001–1.003; *P* < 0.001), TB (HR = 1.002, 95% CI 1.001–1.003; *P* < 0.001), creatinine (HR = 1.008, 95% CI 1.005–1.012; *P* < 0.001), INR (HR = 1.089, 95% CI 1.019–1.163; *P* = 0.012) (Table 2). On multivariable analysis, after adjusting for all competing risk factors, older age (HR = 1.035, 95% CI 1.016–1.055; *P* < 0.001), HE (HR = 3.688, 95% CI 2.371–5.736; *P* < 0.001), alkaline phosphatase (HR = 1.008, 95% CI 1.004–1.011; *P* < 0.001), TB (HR = 1.002, 95% CI 1.001–1.003; *P* = 0.002), creatinine (HR = 1.007, 95% CI 1.001–1.013; *P* = 0.015), INR (HR = 1.114, 95% CI 1.013–1.225; *P* = 0.026) were all independently associated with decreased OS (Table 2).

**Table 2:** Univariable and multivariable Cox proportional hazards analysis for overall survival of acute-on-chronic hepatitis B liver failure

	Univariable analysis			Multivariable analysis		
	HR	95%CI	*P*-value	HR	95%CI	*P*-value
**Clinical parameters**						
Age (years)	1.045	1.030–1.060	<0.001	1.035	1.016–1.055	<0.001
Male gender*	0.903	0.585–1.390	0.647	1.241	0.744–2.069	0.407
Hepatic encephalopathy*	4.610	3.125–6.799	<0.001	3.688	2.371–5.736	<0.001
Liver cirrhosis*	2.930	1.963–4.373	<0.001	1.644	0.948–2.852	0.077
Infection*	2.292	1.573–3.340	<0.001	1.370	0.885–2.12	0.158
Ascites*	2.043	1.357–3.077	<0.001	1.102	0.645–1.884	0.722
Hepatorenal syndrome*	1.834	0.893–3.770	0.099	1.125	0.435–2.906	0.808
**Laboratory parameters**						
White blood cell (10^12^/L)	1.008	0.972–1.046	0.666	0.962	0.904–1.023	0.216
Haemoglobin (g/L)	0.991	0.983–1.000	0.430	1.005	0.994–1.017	0.376
Platelet (10^9^/L)	1.001	0.995–1.007	0.761	1.000	0.993–1.007	0.970
Serum sodium (mmol/L)	0.936	0.908–0.965	<0.001	0.963	0.927–1.001	0.058
ALT (U/L)	1.000	1.000–1.000	0.404	1.000	0.999–1.000	0.155
AST (U/L)	1.000	1.000–1.001	0.030	1.000	1.000–1.001	0.086
Albumin (g/L)	0.969	0.938–1.000	0.053	1.036	0.988–1.087	0.146
Alkaline phosphatase (U/L)	1.002	1.001–1.003	<0.001	1.008	1.004–1.011	<0.001
Total bilirubin (μmol/L)	1.002	1.001–1.003	<0.001	1.002	1.001–1.003	0.002
Creatinine (μmol/L)	1.008	1.005–1.012	<0.001	1.007	1.001–1.013	0.015
INR	1.089	1.019–1.163	0.012	1.114	1.013–1.225	0.026

**NOTE:** ALT, alanine aminotranferase; AST, aspartate aminotranferase; INR, international normalized ratio; MELD, model for end-stage liver disease; HR, hazard ratio; CI, confidence interval.

* Dichotomous values.

### Comparision of overall and conditional survival

**Figure 1:**
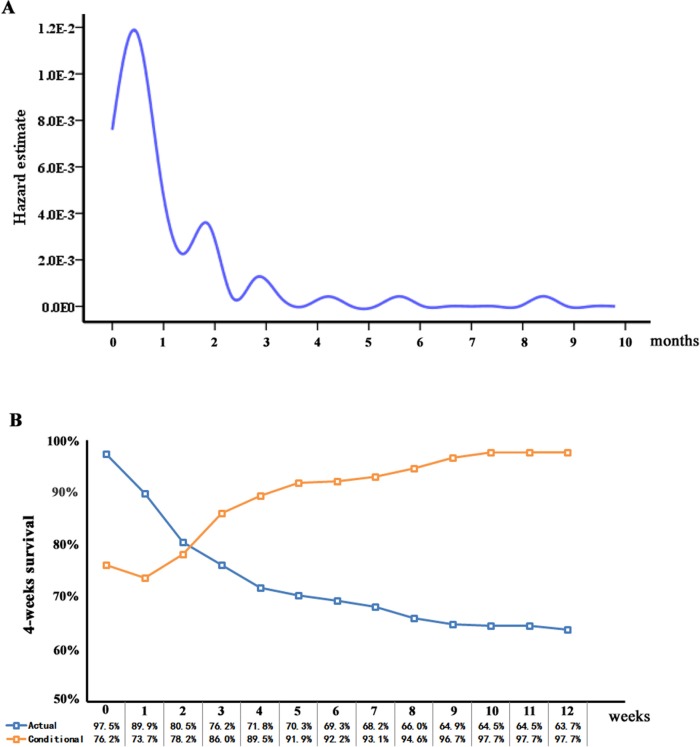
A. Hazard estimate of death in this large multicenter cohort and B. 4-week conditional survival estimate relative to actual survival

When stratified over time, the hazard of death peaked at 2 weeks after diagnosis established and subsequently decreased thereafter (Figure 1). Actual OS at 4 weeks was 71.8% and decreased to 63.7% at 12 weeks. The 4-week CSE at 4 weeks (CS_4_), which means the probability of surviving to 8 weeks after having already survival to week 4 after the date of diagnosis established, was 89.5%. Similarly, the 8-week CS_4_, which means the probability of surviving to 12 weeks after having already survival to week 8, was 94.6% compared with an actual OS 12-week rate of 63.7%. 12-week CS_4_ rates increased over from 76.2% to 97.7% (*P* < 0.001), whereas actual OS deceased over time from 71.8% at 4 weeks to 63.7% at 16 weeks (*P* < 0.001). CSE based on time already survived are summarized in Table 3. The probability of surviving an additional 4 weeks, given that the patient had survived for 1, 3, 5, 7, 9 weeks was 74%, 86%, 92%, 93%, 97%, respectively.

**Table 3:** Proportion of patients with acute-on-chronic hepatitis B liver failure who reach a certain survival time point, given that they have already survived a certain amount of time

Total survival time	If the patient has survived to											
	1 week	2 weeks	3 weeks	4 weeks	5 weeks	6 weeks	7 weeks	8 weeks	9 weeks	10 weeks	11 weeks	12 weeks
1 week												
2 weeks	92.2%											
3 weeks	82.6%	89.5%										
4 weeks	78.1%	84.7%	94.6%									
5 weeks	73.7%	79.9%	89.2%	94.3%								
6 weeks	72.2%	78.2%	87.4%	92.4%	98.0%							
7 weeks	71.0%	77.0%	86.0%	90.9%	96.5%	98.4%						
8 weeks	69.9%	75.8%	84.7%	89.5%	94.9%	96.9%	98.4%					
9 weeks	67.7%	73.4%	81.9%	86.6%	91.9%	93.8%	95.2%	96.8%				
10 weeks	66.5%	72.1%	80.6%	85.2%	90.3%	92.2%	93.7%	95.2%	98.3%			
11 weeks	66.2%	71.7%	80.1%	84.7%	89.8%	91.7%	93.1%	94.6%	97.8%	99.4%		
12 weeks	66.2%	71.7%	80.1%	84.7%	89.8%	91.7%	93.1%	94.6%	97.8%	99.4%	100.0%	
13 weeks	65.4%	70.9%	79.2%	83.7%	88.8%	90.6%	92.1%	93.5%	96.7%	98.3%	98.9%	98.9%

**Figure 2:**
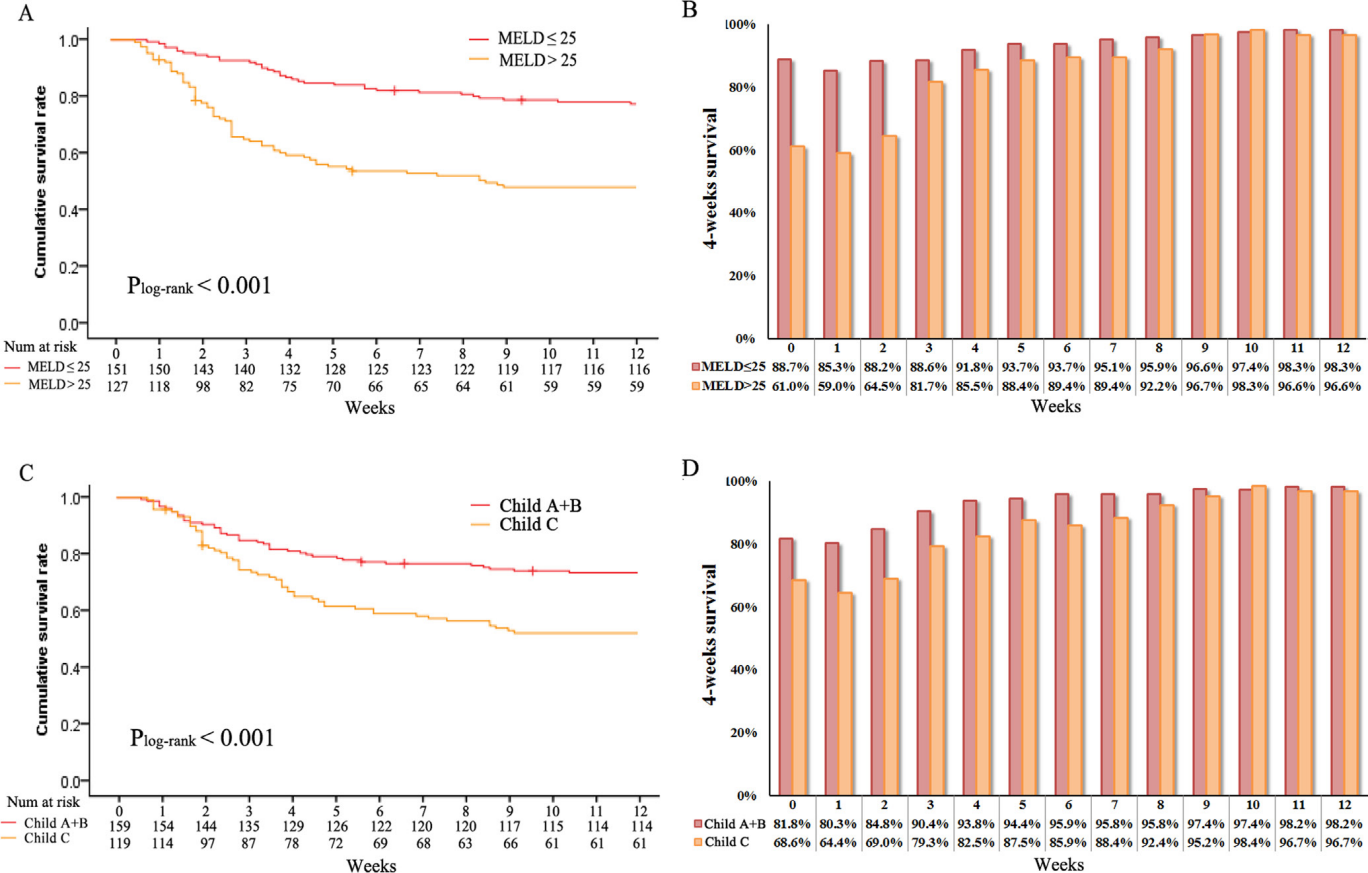
Overall survival stratified by **A.** MELD score (log-rank *P* < 0.001), **C.** Child score (log-rank *P* < 0.001) and conditional survival estimates stratified by **B.** MELD score and **D.** Child score.

As shown in the Kaplan-Meier analysis, OS worsened with increasing MELD score and Child grade C (both log-rank *P* < 0.001, Figure 2A and 2C). For example, 8-week actual OS for patients with MELD ≤ 25 was 81% and decreased with worsening extent of disease: 52% for MELD > 25. The calculated CS_4_ exceeded the actual OS for all corresponding MELD score (Figure 2B). Furthermore, this difference was more significant for those patients who were initially predicted to have poorer prognosis. For example, patients with MELD > 25 had a CS_4_ of 92% at 8 weeks compared with an actual OS of 51% at 12 weeks (Δ41%). Similarly, patients with Child grade C had a CS_4_ of 92% at 8 weeks compared with an actual OS of 56% at 12 weeks (Δ36%) (Figure 2D). Conversely, patients with “favorable” characteristics had smaller differences between actual OS and CSE. Specifically, patients with MELD ≤ 25 had an actual 12-week OS of 78% compared to a 8-week CS_4_ of 96% (Δ18%). Similarly, patients with Child grade A and B had a 8-week CS_4_ of 96% compared with an actual OS of 74% at 12 weeks (Δ22%).

**Figure 3:**
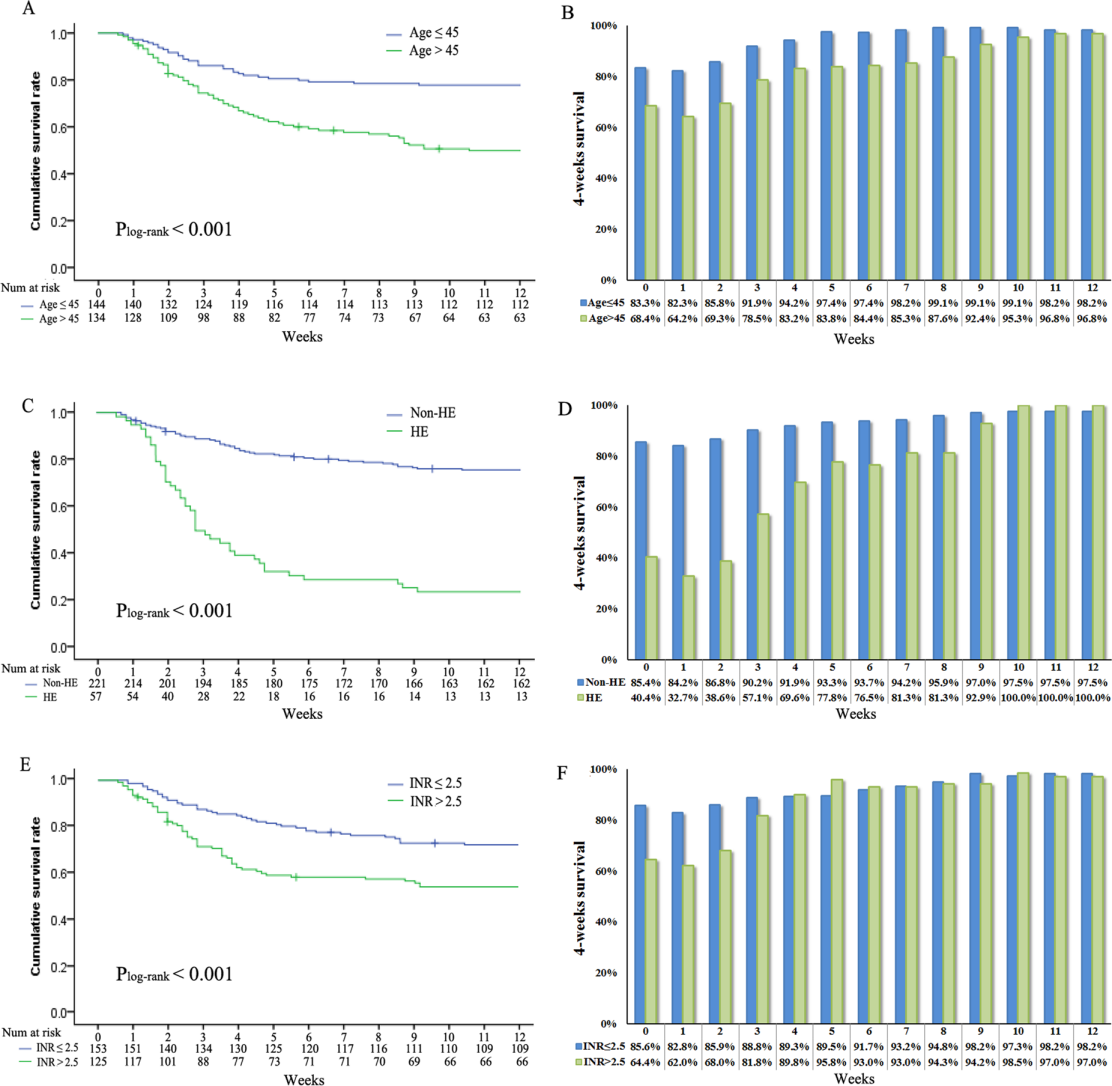
Overall survival stratified by **A.** age (log-rank *P* < 0.001), **C.** hepatic encephalopathy (log-rank *P* < 0.001), **E.** international normalized ratio (log-rank *P* < 0.001) and conditional survival estimates stratified by **B.** age, **D.** hepatic encephalopathy and **F.** international normalized ratio.

**Figure 4:**
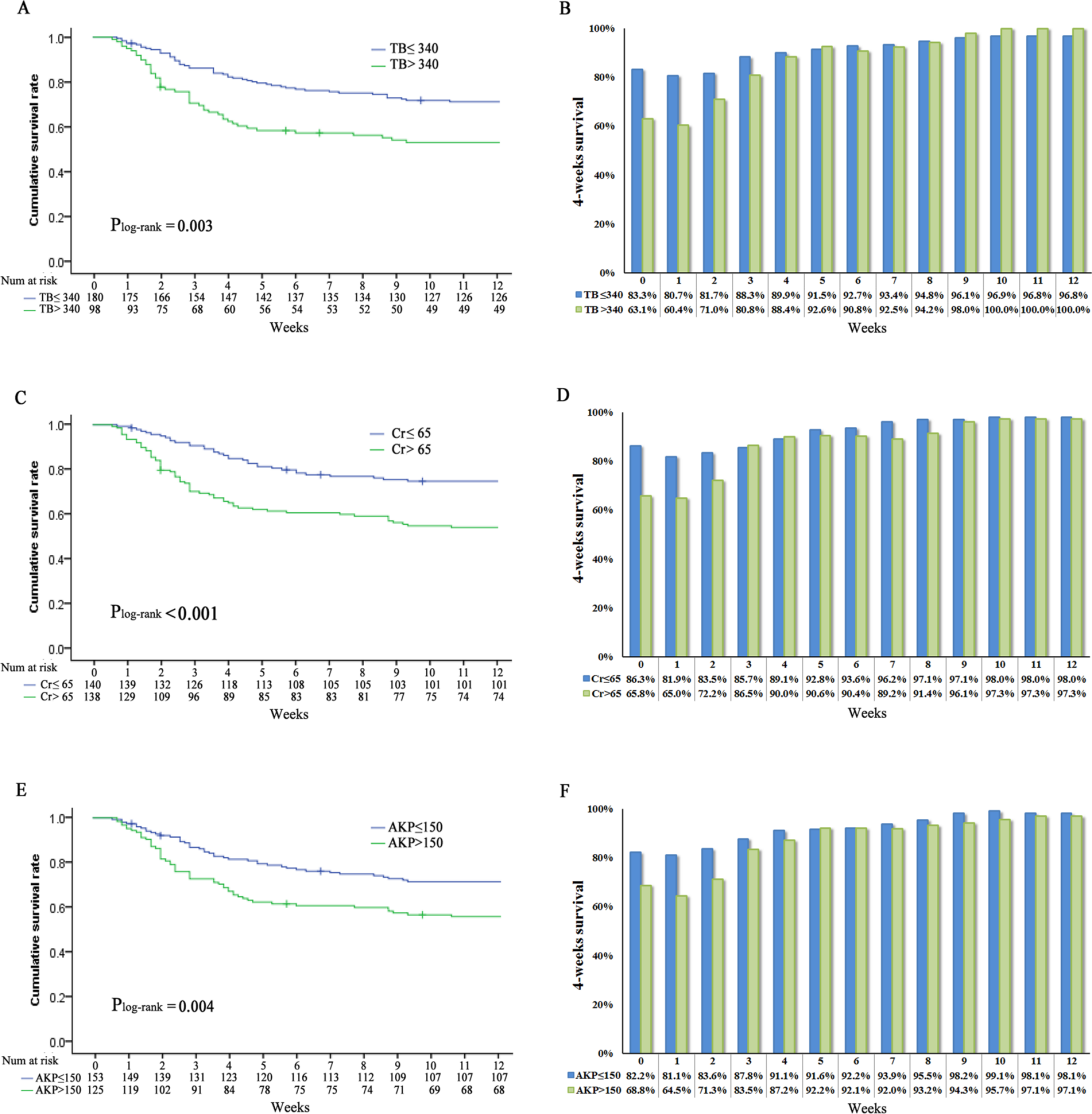
Overall survival stratified by **A.** total bilirubin (log-rank *P* = 0.003), **C.** creatinine (log-rank *P* < 0.001), **E.** alkaline phosphatase (log-rank *P* < 0.004) and conditional survival estimates stratified by **B.** total bilirubin, **D.** creatinine and **F.** alkaline p hosphatase.

CS_4_ also was stratified by different clinical and laboratory variables of known prognostic importance, such as age, HE and INR. CS_4_ increased over time among patients younger than 45 years old (83%–98%, Δ15%) and older than 45 years (68%–97%, Δ29%; *P* < 0.001) (Figure 3A, 3B). The differences in 8-week CS_4_ over time were more pronounced among patients with worse prognostic features. Smaller changes over time in 8-week CS_4_ were seen in patients without HE (85%–97%, Δ12%) compared with patients with HE (40%–100%, Δ60%; *P* < 0.001) (Figure 3C, 3D). Similarly, patients with INR > 2.5 (64% - 97%, Δ33%) had larger difference in 8-week CS_4_ compared with patients with INR ≤ 2.5 (86%–98%, Δ12%) (Figure 3E, 3F). These patterns were also observed in 8-week CS_4_ differences between patients with MELD ≤ 25 (89%–98%, Δ9%) and MELD > 25 (61%–97%, Δ36%), as well as between patients with Child grade A and B (82%–98%, Δ16%) and Child grade C (69%–97%, Δ28%) (all *P* < 0.001). Similarity, same trends had been found in the remaining prognostic factors (TB, creatinine, alkaline phosphatase; Figure 4A–4F).

## DISCUSSION

ACHBLF is an aggressive critical condition with high short-term mortality risk [1, 5, 25]. There is still no easy-to-use mortality risk prediction tool derived from a population-based prospective cohort study [4]. To our knowledge, this study is the first to provide strong support for the use of CSE in the prediction of short term mortality in ACHBLF patients as these estimates have a stronger dynamic predictive ability compared with traditional OS. With wide-ranging survival estimates, static prediction of OS based on data around the time of diagnosis may not be accurate as patients had survived for a period of time.

In this large prospective multicenter study, the actual OS at 2, 4, 6, 8, 12 weeks were 80.5%, 71.8%, 69.3%, 66.0% and 63.7%, respectively. However, we had also noted that the hazard of death did not remain constant over time. Rather, the probability of CSE increased over time based on survival time already accumulated, providing support for the concept that CSE may be a better estimate of prognosis as survival time accrues. In fact, on average, CSE increased over time and were significantly higher than actual OS, which was consistent with the trends from other research groups [13–14, 17]. Furthermore, the magnitude in difference between CSE and OS were highest among patients with worse prognostic features.

Depending on the patient and liver-specific factors, including age, HE, INR, MELD, Child score, CSE may provide a more accurate and clinically relevant patient-specific survival estimate. However, these differences were not uniform across all patients. Patients with worse prognostic features had a higher increase in CSE based on actual time survived. For example, patients without HE showed only a 12% increase in 8-week CS_4_ compared with a 60% increase in patients with HE and the critical role of HE in ACHBLF has been recently been highlighted [26]. Similarly, patients with MELD > 25 showed a 36% increase in 8-week CS_4_ compared with a 10% increase in patients with MELD ≤ 25. As such, CSE should be favored and used in this instance, particularly in high-risk patients.

Compared with existing scoring systems that we had described, including CLIF Consortium Organ Failure score (CLIF-C OFs), CLIF-SOFA, ANN, ALPH-Q, LRM, MELD et al. [7–11, 27], current CSE do not require physicians to perform complex calculations, but rather, they can easily extract the estimated risk and the impact on risk when various risk factors are added or removed. Furthermore, the CSE enable physicians to more easily engage with a target patient in an individual discussion of risk and thus enhance risk communication [28].

There are, however, some limitations of this study. First, because the present study is a multicenter analysis, there may have been selection bias in the cohort. However, the multicenter nature of the current study indeed provides support to the generalizability of our results. Second, current CSE only rendered the data of the first 3 months. As a critical disease, death was not uncommon in the early stages and paying more attention to short-term mortality will allow a better future assessment of long-term survival. Finally, external validation of this CSE in a more diverse patient population, preferentially in the clinical setting, is necessary.

In summary, this easy-to-use CSE should permit physicians to assess the individual risk of ACHBLF patients and facilitate risk communications between physicians and patients.

## MATERIALS AND METHODS

### Study population

We prospectively enrolled patients from three separate medical centers (the First Affiliated Hospital of Wenzhou Medical University, Ningbo No. 2 Hospital and Tianjin Infectious Disease Hospital, from December 2010 to December 2013) with the same medical record systems. The start date of the follow-up was the date of diagnosis of ACHBLF. All patients were followed up for at least 3 months. Written informed consent was obtained from each patient included in the study and the research protocol of the study was approved by the Ethics Committee of the First Affiliated Hospital of Wenzhou Medical University, Ningbo No. 2 Hospital and Tianjin Infectious Disease Hospital.

### Inclusion and exclusion criteria

ACLF was diagnosed according to the recommendation of the APASL [2]. In brief, ACLF is defined as acute hepatic insult manifesting as jaundice and coagulopathy, complicated within 4 weeks by ascites or encephalopathy in a patient with previously diagnosed or undiagnosed chronic liver disease. ACHBLF is defined as ACLF caused solely by HBV. Patients who meet the following criteria were excluded: 1) infected and/or co-infected with non-HBV; 2) autoimmune diseases; 3) alcohol abuse; 4) past or current HCC; 5) toxic caused liver disease; 6) pregnancy; 7) liver transplantation previously. Once diagnosis of ACHBLF, the cares that provided to the included patients at three centers were same, and in accordance with the Asia-Pacific consensus recommendations [2]. This routinely included oral antiviral agents, absolute bed rest, energy supplements and vitamins, intravenous drop infusion albumin, maintenance water, electrolyte and acid-base equilibrium and prevention and treatment complications, etc.

### Data collection

A detailed history of all the patients was taken when they were in hospital. Patient medical history was recorded upon admission and every 2-weeks during follow-up. Patient characteristics were detected within the first 24 hours after the established diagnosis of ACHBLF. Confirmatory physical examination, laboratory tests and abdominal ultrasound scanning were performed.

Clinical parameters included age, gender, body mass index, blood pressure, hepatic encephalopathy (HE), liver cirrhosis (LC), ascites and hepatorenal syndrome (HRS). The HE grade was re-classified into 0: non-HE, 1: mild (grade 1–2) and 2: severe (grade 3–4) according to West-Haven criteria [29]. LC was defined by the following combined parameters: (1) a score greater than 2 according to the aspartate aminotransferase (AST) to platelet ratio using the formula: [AST/upper limit of normal]/platelet count (×10^9^/L) × 100 [30], (2) ultrasonographic evidence of a small sized liver with and without splenomegaly/ascites, and (3) an albumin level less than 35 g/L without other identifiable causes of hypoalbuminemia such as renal loss or gastrointestinal loss. The detection of ascites included history, physical examination, abdominal ultrasound, and laboratory assessment of liver function, renal function, serum and urine electrolytes. We re-classified ascites grade into 0: non-ascites, 1: mild (grade 1), 2: moderate to severe (grade 2–3) [31]. HRS was defined as low glomerular filtration rate, as indicated by serum creatinine of >1.5 mg/dL or 24-h creatinine clearance <40 ml/min, without the presence of chronic kidney diseases [32].

Laboratory parameters including alanine aminotransferase, AST, total bilirubin (TB), albumin, platelet count, hemoglobin, serum creatinine, international normalized ratio (INR), serum sodium and potassium. HBV serologic markers were collected for each patient (Abbott, AXSYM). Serum HBV DNA was measured by quantitative PCR assay (Roche Amplicor, limit of detectability of 100 IU/ml) after admission. Hepatitis C virus antibody and human immunodeficiency virus antibody were detected using ELISA (IEGAN, Freedom evolyzer/150). Antinuclear antibody was evaluated using indirect immunofluorescence and soluble liver antigen/liver pancreas antigen, anti-liver/kidney microsomal antibody Type 1 and anti- liver cytosol antibody Type 1 were evaluated using immunoblot analysis (Euroimmun, Lubeck, Germany).

### Scoring systems and prognostic models

Child score, which included HE, PT, ascites, TB and serum albumin, was assessed according to the standard criteria [33]. MELD score were calculated according to the Malinchoc formula: R = 9.57 × ln(creatinine [mg/dL]) + 3.78 × ln(bilirubin [mg/dL]) + 11.2 × ln(INR) + 6.43 × (aetiology: 0 if cholestatic or alcoholic, 1 otherwise) [27].

### Follow-up

Patients were followed up for every 2 weeks for 3 months when the diagnosis was established, every 1 month thereafter for a total of 1 year and every 3 months thereafter. Information on death was obtained either from the patient's social security death index, outpatient medical records, or notifications from the family of the deceased. The deadline of follow-up time was March 1, 2015.

### Statistical analysis

The Kolmogorov-Smirnov test was applied to determine whether sample data were likely to be derived from a normal distribution population. Continuous variables of normal and skewed distribution are expressed as mean ± standard deviation and median (interquartile range), respectively. Categorical values were expressed by absolute and relative frequencies. Differences in variables were analyzed using Student *t*-tests (for normally distributed data) or Wilcoxon's Sign Rank Test (for skewed distributed data). The Chi-square test or the Fisher's exact test was used for categorical data as appropriate. OS estimates for the entire study population were generated using the Kaplan-Meier method calculated from the date of diagnosis to the date of last follow-up or death. The association of relevant variables with OS was assessed using Cox proportional hazards models. Variables in the univariate Cox regression analysis were progressed to a multivariate analysis using forward stepwise selection. According to the previous findings [7–9], the risk of death is greatest within the first three months after the date of diagnosis established. So, 12-week was chosen as the main cutoff of timeline. CSE was calculated as the probability of survival for an additional 4 week (CS_4_), given that the patient had survived for 1, 2, 3, 4, or more weeks, calculated as CS_4_ = OS_(X+4)_/OS_(X)_. For example, 4-week CSE among patients who have survived 2 weeks from the date of diagnosis is calculated by dividing the 6-week OS rate by the 2-week OS rate. Changes in CS_4_ over time were assessed using linear regression. For all analyses, a *P* value of < 0.05 was considered statistically significant. Statistical analysis was performed using SPSS version 20.0 (SPSS, Chicago, IL, USA).

## SUPPLEMENTARY TABLE


