# Supplementary material for: Conditional survival estimate of acute-on-chronic hepatitis B liver failure: A dynamic prediction based on a multicenter cohort
Source: Oncotarget. 2015 Jul 15;6(27):23261–71. doi: 10.18632/oncotarget.4666 (PMC4695116; doi:10.18632/oncotarget.4666)
Supplement: Supplementary file 1 [file oncotarget-06-23261-s001.pdf]

SUPPLEMENTARY TABLE

Supplementary Table S1. Characteristics of patients with acute-on-chronic hepatitis B liver failure

Variable	Total (*n* = 278)
**Clinical parameters**	
Age (years)	45.8 ± 13.2
Male gender No.(%)*	215 (77.3%)
Hepatic encephalopathy No.(%)*	57 (20.5%)
Liver cirrhosis No.(%)*	127 (45.6%)
Infection No.(%)*	101 (36.6%)
Ascites No.(%)*	156 (56.1%)
Hepatorenal syndrome No.(%)*	15 (5.4%)
**Laboratory parameters**	
White blood cell (10^{12}/L)	7.37 ± 4.65
Haemoglobin (g/L)	125.08 ± 22.53
Platelet (10^9/L)	46.1 ± 30.24
Serum sodium (mmol/L)	136.22 ± 5.34
ALT (U/L)	591.49 ± 647.68
AST (U/L)	516.1 ± 668.87
Albumin (g/L)	30.85 ± 5.75
Alkaline phosphatase (U/L)	153.26 ± 58.81
Total bilirubin (μmol/L)	294.1 ± 162.52
reatinine (μmol/L)	74.19 ± 41.79
INR	2.65 ± 1.5
**Scoring systems**	
MELD score	25.49 ± 7.98
Child score	9.11 ± 1.58

NOTE: ALT, alanine aminotranferase; AST, aspartate aminotranferase; INR, international normalized ratio; MELD, model for end-stage liver disease.
*Dichotomous values.
